# Technical advances in proteomics: new developments in data-independent acquisition

**DOI:** 10.12688/f1000research.7042.1

**Published:** 2016-03-31

**Authors:** Alex Hu, William S. Noble, Alejandro Wolf-Yadlin

**Affiliations:** 1 Department of Genome Sciences, University of Washington, Seattle, WA, 98109, USA

**Keywords:** proteomics, data-independent acquisition, mass spectrometry

## Abstract

The ultimate aim of proteomics is to fully identify and quantify the entire complement of proteins and post-translational modifications in biological samples of interest. For the last 15 years, liquid chromatography-tandem mass spectrometry (LC-MS/MS) in data-dependent acquisition (DDA) mode has been the standard for proteomics when sampling breadth and discovery were the main objectives; multiple reaction monitoring (MRM) LC-MS/MS has been the standard for targeted proteomics when precise quantification, reproducibility, and validation were the main objectives. Recently, improvements in mass spectrometer design and bioinformatics algorithms have resulted in the rediscovery and development of another sampling method: data-independent acquisition (DIA). DIA comprehensively and repeatedly samples every peptide in a protein digest, producing a complex set of mass spectra that is difficult to interpret without external spectral libraries. Currently, DIA approaches the identification breadth of DDA while achieving the reproducible quantification characteristic of MRM or its newest version, parallel reaction monitoring (PRM). In comparative *de novo* identification and quantification studies in human cell lysates, DIA identified up to 89% of the proteins detected in a comparable DDA experiment while providing reproducible quantification of over 85% of them. DIA analysis aided by spectral libraries derived from prior DIA experiments or auxiliary DDA data produces identification and quantification as reproducible and precise as that achieved by MRM/PRM, except on low‑abundance peptides that are obscured by stronger signals. DIA is still a work in progress toward the goal of sensitive, reproducible, and precise quantification without external spectral libraries. New software tools applied to DIA analysis have to deal with deconvolution of complex spectra as well as proper filtering of false positives and false negatives. However, the future outlook is positive, and various researchers are working on novel bioinformatics techniques to address these issues and increase the reproducibility, fidelity, and identification breadth of DIA.

## Introduction

For the last 15 years, liquid chromatography-tandem mass spectrometry (LC-MS/MS)-based proteomics has provided broad detection and relative quantification—through chemical or metabolic labeling—of thousands of proteins across a variety of biological samples using a data-dependent acquisition (DDA) strategy ^1– 4^. In recent years, alternative LC-MS/MS targeted acquisition strategies, such as multiple-reaction monitoring (MRM) ^5– 7^ and parallel reaction monitoring (PRM) ^8^, have provided precise and reproducible absolute quantification of up to hundreds of proteins. The ultimate goal of proteomics is the development of acquisition strategies that have both the breadth of DDA and the precision of MRM/PRM to provide reproducible identification and quantification of every protein in any biological sample. Although no single acquisition strategy can yet achieve this goal, recent advances in hardware and software show that a recently resurfaced strategy ^9^, data-independent acquisition (DIA), may provide a viable path to this goal ^10^. Below is a discussion of DDA, MRM/PRM’s shortcomings, DIA’s circumvention of these shortcomings, current software to analyze DIA spectra, and efforts to further improve DIA analysis.

**Figure 1.**
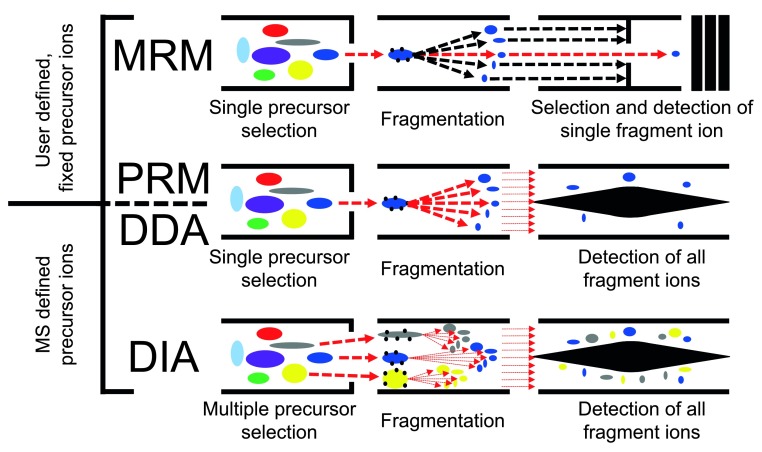
A cartoon schematic of how peptides are isolated, fragmented, and analyzed by a mass spectrometer working in data-dependent acquisition (DDA), multiple reaction monitoring (MRM), parallel reaction monitoring (PRM), or data-independent acquisition (DIA) modes. In DDA, MRM, and PRM, single precursor ions are isolated, fragmented, and analyzed in an MS2 scan by the mass spectrometer. In DDA mode, the precursor ions are chosen by the instrument on the basis of abundance. In MRM and PRM, the precursor ions to be analyzed are fixed by the user. DIA is different form the methods above in that all precursor ions within a selected mass range are isolated, fragmented, and analyzed in a single MS2 scan. MS1, scan in which the peptide ions entering the mass spectrometer at a given time are identified; MS2, scan in which the fragments of all (or some) of the peptides that are in the mass spectrometer at a given time are identified.

All LC-MS/MS methods discussed in this article are bottom-up proteomics: Proteins are enzymatically digested into peptides which then are separated using high-performance liquid chromatography (HPLC), ionized, isolated, fragmented, and detected in the mass spectrometer as they elute from the HPLC. HPLC delivers peptides into the mass spectrometer for a period of time (tens of minutes to a few hours, depending on the application), separating the peptides according to their physicochemical characteristics ^4, 11^, thus increasing sample coverage. In LC-MS/MS methods, three events occur in the mass spectrometer: (a) ionization: peptides elute into the mass spectrometer from the HPLC and are ionized; (b) MS1 scan: the abundance and mass-to-charge ratios (m/z) of all ions eluting at a given time are measured; and (c) MS2 scan: some or all detected ions are fragmented, and the abundances and m/z’s of the fragments are measured and recorded. Different LC-MS/MS methods vary in how ions are selected and measured in the MS2 scan. Figure 1 shows a cartoon schematic of how peptides are isolated, fragmented, and analyzed by a mass spectrometer working on DDA, MRM, PRM, or DIA modes.

**Figure 2.**
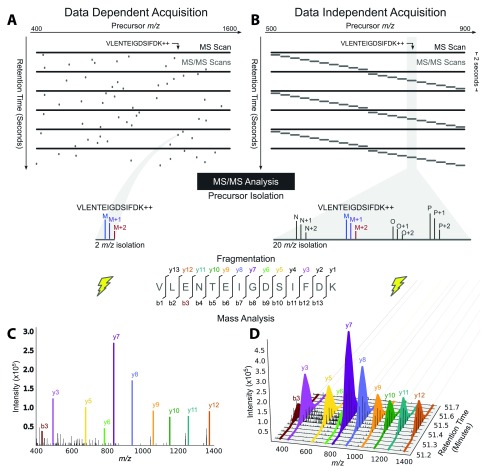
Tandem mass spectrometry (MS/MS) analysis in data-dependent acquisition and data-independent acquisition ^50^. ( **A**) Data-dependent acquisition (DDA) acquires MS/MS scans with narrow isolation windows centered on peptide precursors detected in an MS scan over a wide range of masses: 400 to 1,600 mass-to-charge ratio (m/z) here. ( **B**) Data-independent acquisition (DIA) acquires MS/MS scans with wide isolation windows that do not target any particular peptide precursor. Instead, the scans are arranged side-by-side to collectively cover a desired precursor m/z range (500 to 900 m/z here) comprehensively, and several precursors are fragmented together in a single MS2 event (four here: identified peptide M and peptides N, O, and P). ( **C**) Fragment ion information for the peptide precursor VLENTEIGDSIFDK++ is present in a single MS/MS spectrum in a DDA analysis, ( **D**) but it can be extracted over time from DIA data and used for quantification owing to the repetitive MS/MS sampling cycle of DIA. Adapted with permission from Egertson *et al.* ^46^.

In DDA, a subset of the most abundant ions reaching the mass spectrometer detector during an MS1 scan are individually isolated and fragmented in sequential MS2 scans ( Figure 1 and Figure 2A), and each MS2 scan ( Figure 2C) can be analyzed with a database search algorithm ^1, 4^. Currently, most instruments can perform a DDA cycle with one MS1 scan and 10 MS2 scans within 2 seconds. DDA typically yields thousands of protein identifications. Unfortunately, irreproducibility and imprecision are fundamental to DDA’s design; if too many peptide species co-elute and appear in a single MS1 scan, then DDA stochastically samples only the most abundant peptides and misses the rest. This approach diminishes reproducibility and prevents the measurement of low-abundance peptides ^9^. Additionally, to survey as many peptides as possible, DDA deliberately samples each peptide species only once or twice, preventing precise absolute quantification that requires multiple measurements per peptide. DDA analysis has been used for a variety of studies, including the characterization of epidermal growth factor receptor (EGFR) signaling networks ^12, 13^, the characterization of the proteome on different mouse organs ^14, 15^, identification of protein interaction partners ^16– 18^, and description of the role of viral infections in modulating host proteomes ^18– 21^, which had been thoroughly covered in a special issue of the journal *Proteomics* ^22^. In spite of its flaws, DDA’s flexibility, breadth of detection, and the simplicity of its setup and analysis, make DDA the preferred LC-MS/MS method among the wider scientific community. Additionally, DDA allows relative quantification of peptides between selected samples through a variety of chemical labeling schemes—e.g., isotopic (stable isotope labeling by amino acids in cell culture, or SILAC) ^23^ or isobaric (isobaric tags for relative and absolute quantitation, or iTRAQ) ^24^ labeling.

Alternatively, the targeted methods MRM and PRM avoid the imprecision and irreproducibility of DDA by focusing MS2 scans on only a small set of predetermined and previously identified peptides. Instead of selecting the top *n* precursors in an MS1 scan for further fragmentation in MS2 scans, these methods select only precursors and fragments with the m/z and elution time that match a pre-specified peptide of interest. The knowledge of a peptide’s elution time, MS1 m/z value, and robustly detectable fragments is determined prior to the MRM/PRM experiments by previous DDA identification or MS/MS measurement of its synthetic version in a simplified background or both. In MRM, for each MS1 scan, a subset of fragment ions is measured in the subsequent MS2 scan ^25^, whereas in PRM, all of the fragment ions are measured ^8^. In both, the same precursors are selected and fragmented multiple times to acquire more precise quantification of fewer peptides, compared with DDA. MRM was developed first and has been shown to robustly quantify tens of blood plasma biomarkers of low abundance across laboratories toward clinical use ^6^ and tens of low-abundance transcription factors and kinases in human cells ^7^. Currently, the Clinical Proteomics Tumor Analysis Consortium (CPTAC) ^26^ database hosts a collection of 679 MRM assays for human proteins. PRM, which succeeded MRM, outperforms it in terms of throughput and absolute quantification thanks to the use of high-resolution spectrometers capable of parallel fragment analysis ^8, 27^. PRM is still evolving; however, it has already been used successfully to address difficult proteomics problems in human health, such as the role of low-abundance polyubiquitin chains ^28^ in Parkinson’s disease ^29^, and in plant biology to monitor the degradation of low-abundance peptides in *Arabidopsis thaliana* ^30^.

DIA is like MRM/PRM in that it repeatedly samples the same peptides for more precise quantification, but it differs from them and DDA by dispensing with the isolation of individual peptide species and instead repeatedly selecting mixtures of peptide species within large, pre-specified mass ranges ( Figure 1 and Figure 2B) for MS2 scans. DIA is therefore guaranteed to sample all peptides within the selected mass ranges, allowing for the identification of all sufficiently abundant peptides within them if the resulting spectra are properly interpreted ^10^.

Proper interpretation of DIA data is currently problematic because the complex MS2 scans contain mixtures of peptides and therefore are more difficult to analyze. Fortunately, recent developments in bioinformatics software have adequately overcome this DIA issue, so that DIA now closely matches DDA in the number of peptide identifications while still allowing precise quantification of most of them. Quantification relies on comparing DIA spectra to sets of annotated and refined peptide-MS2 spectrum matches from DDA experiments (or the same DIA experiment in DIA-Umpire’s algorithm) called spectral libraries that show accurate, empirically determined fragmentation patterns for each peptide in the library. However, DIA is currently unable to match the precision of MRM or PRM in measuring very low-abundance peptides, likely because their signals are dwarfed by those from abundant co-eluting peptides. A brief comparison of DDA, DIA, and MRM/PRM with respect to precision of quantification, breadth of identification, ease of setup and analysis, and reproducibility, is shown in Table 1.

**Table 1.** Advantages and disadvantages of data-dependent acquisition, parallel reaction monitoring/multiple reaction monitoring, and data-independent acquisition methods.

Method	Instrument setup	Ease of data analysis	Precision of peptide quantification	Reproducibility of peptide identification	Breadth of peptide identification
DDA	Easiest Requires user selection of precursor m/z range and frequency of precursor scans. Is the default mode of use on most commercial instruments.	Easiest Many convenient and comprehensive pipelines for the analysis of DDA spectra have been developed over more than 20 years ^48, 51, 52^.	Low/Moderate/High Spectral counts (low), isobaric-tag labels (moderate), or SILAC (high) can be used for relative quantification of protein abundance across samples. Hard to use for absolute quantification.	Lowest Run-to-run peptide identification overlap for a given sample is around 60% ^53^.	Highest Samples and identifies a single time as many peptides as can be individually isolated.
PRM/MRM	Hardest Requires prior identification of peptides and, in MRM, selection of reproducible fragments that do not exhibit interference ^54^.	Moderate A few pipelines have been developed over the past few years but require some manual curation to identify and quantify fragment chromatograms ^54^.	Highest Provides good relative peptide quantification and can be coupled with heavy labeled reference peptide for absolute quantification. Most sensitive method because of high signal-to-noise ratio ^54^.	Highest Run-to-run peptide identification overlap for a given sample is more than 85% ^54^.	Low Repeatedly samples and identifies a small set of pre-specified peptides ^54^.
DIA	Easy Requires user selection of precursor m/z windows for MS1 and MS2 scans.	Hardest Requires multiple steps from multiple experiments to compile spectral libraries, with more parameters to choose in recently developed, not-yet- established pipelines.	Moderate/High Similar to PRM/MRM but more vulnerable to variation caused by interference from other peptides ^32– 37, 39– 45, 47, 50– 54^.	High Similar to PRM/MRM but more vulnerable to variation caused by interference from other peptides ^32– 37, 39– 45, 47, 50– 54^.	High Repeatedly samples every peptide within pre-specified m/z windows and identifies those whose signals can be successfully deconvolved ^54^.

DDA, data-dependent acquisition; DIA, data-independent acquisition; MRM, multiple reaction monitoring; MS1, scan in which the peptide ions entering the mass spectrometer at a given time are identified; MS2, scan in which the fragments of all (or some) of the peptides that are in the mass spectrometer at a given time are identified; m/z, mass-to-charge ratio; PRM, parallel reaction monitoring; SILAC, stable isotope labeling by amino acids in cell culture.

**Figure 3.**
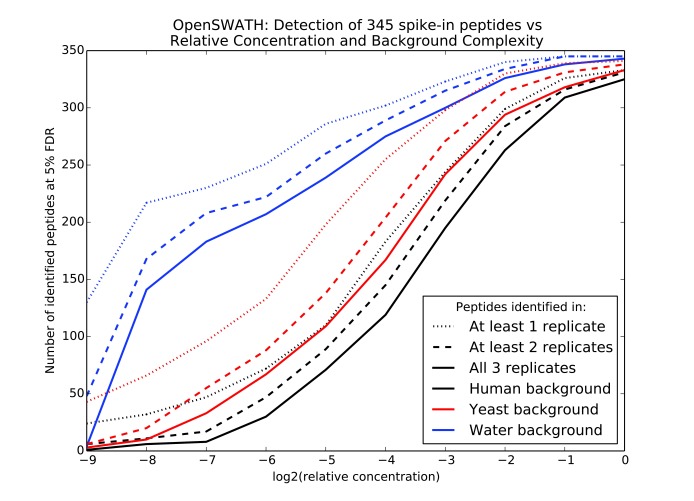
Effect of peptide concentration and sample complexity on identification sensitivity ^31^. Varying concentrations of 345 synthetic peptides were spiked into three sample backgrounds, subjected to data-independent acquisition (DIA), and analyzed by OpenSWATH. Lines show the number of spike in peptides identified at a 5% false discovery rate (FDR) in the different samples.

The complexity of MS2 spectra greatly impacts the sensitivity of the downstream analyses and therefore must be considered when planning a DIA experiment. Two main variables determine the complexity and interpretability of the spectra: the number of proteins present in the sample and the precursor m/z window widths from which ions are isolated and fragmented. The effect of the number of proteins present is shown in Figure 3, which describes the results of a study in which 345 synthetic peptides were spiked into water, yeast lysate, or human lysate backgrounds in concentrations varying from 30 to 0.058 fmol/µL and then analyzed via relatively wide precursor isolation windows of 25 m/z ^31^ (see Supplementary material). The sensitivity of peptide detection decreases with the protein complexity of the organism, where the yeast lysate is more complex than the water background and the human lysate is more complex than the yeast lysate, with an average of 32,993 unique human, trypsin-digested peptide ions possibly falling within each 25-m/z window compared with the 7,287 yeast peptide ions. Sensitivity and precision may be increased by shortening the width of the precursor isolation window, thereby decreasing the number of peptides represented in each MS2 spectrum. However, this decreases coverage across the total precursor m/z range and therefore the number of peptides to which the analysis may be sensitive. Therefore, experimentalists must balance the effects of sample complexity, isolation window width, and desired coverage on sensitivity.

The main reason for the decrease in sensitivity is the increased likelihood of fragment ion interference in complex spectra, which occurs when multiple co-eluting peptides share a fragment ion peak. Interference undermines the elution profile correlation between a peptide’s fragments and the fragments’ correlation to spectral libraries on which many DIA analysis methods rely. In an analysis of synthetic peptides spiked into human urine, SWATHProphet ^32^, a software tool further described below, estimated that at least 24% of confidently identified peptides showed evidence of fragment ion interference that increased the variance of their quantification. This percentage is likely much higher in unidentified peptides and this is the likely cause of the peptides’ invisibility to the software. Therefore, further progress in the interpretation of DIA spectra should circumvent the problem of interference, which SWATHProphet ^32^ has begun to do by identifying and disregarding fragments affected by interference.

## Data-independent acquisition strategies

In all DIA methods, each MS2 scan contains fragments from every peptide within one or more pre-specified precursor m/z windows. Each window is repeatedly sampled so that each peptide is fragmented multiple times. The earliest and most common DIA method is a sequential sampling strategy ^9, 33^ ( Figure 2B), in which an m/z range covering most peptides of interest is split into a sequence of non-overlapping windows, usually of equal but sometimes of variable size depending on the m/z distribution of the peptides of interest. For each window in the sequence, all of the precursors falling into that window are fragmented together and measured in an MS2 scan. The machine repeats the sequence throughout the full HPLC elution gradient. The time needed to complete the traversal of the sequence is on the order of a few seconds, such that every peptide can be sampled at least a few times during its elution.

An alternative DIA method, MSX ^34^, incorporates multiplexing and an element of randomness to the sequential sequencing to increase precision in associating a precursor ion to its fragments. However, this method is compatible only with selected mass spectrometers in which software controllers have been modified to accept random sampling of mass range windows. The precursor m/z windows are smaller and more numerous than in the sequential method, but each MS2 scan is multiplexed. Each scan contains fragmented precursors from multiple, randomly chosen non-contiguous windows, such that the precursors span the same total m/z length in each MS2 scan. Post-processing of the MS2 spectra solves a system of linear equations to infer from which smaller precursor window each fragment ion peak originated. The resulting de-multiplexed MS2 scans allow a modest increase in peptide identifications compared with scans produced from non-random, contiguous windows.

## Computational analysis of spectra

Although traditional algorithms for identifying peptides from DDA spectra can be applied to DIA spectra analysis, these algorithms are not appropriate for DIA for two reasons: they incorrectly assume that each MS2 scan contains fragments from just one peptide, and they ignore the dynamic pattern of elution profiles in DIA spectra. Consequently, three main classes of computational algorithms have emerged to specifically analyze DIA data that accommodate the complexity and time variation in DIA spectra. The first two classes are for untargeted, discovery-based identification, and the third is for precise quantification of previously identified peptides from spectral libraries. The main challenge of the algorithms below is controlling the false discovery rate among the identified peptides while identifying all (or most) real peptides in the sample of interest.

### Deconvolution of MS2 scans over time followed by database search

These methods use a pre-processing step that deconvolves DIA MS2 scans into multiple pseudo-spectra, each containing the fragments of only a single peptide species in the mixture. The intensities of different fragments of the same peptide species should correlate over elution time, and the pre-processing step uses this correlation to assign fragment ions from MS2 scans to their intact peptide species in MS1 scans. These pseudo-spectra then can be searched by using a traditional DDA database search method.

DIA-Umpire ^35^ and DeMux ^36^ are two strategies that take this approach. They differ in the specific algorithms used to group ions and compile them into deconvolved spectra. DIA-Umpire tends to work better because it considers isotope peak distributions in MS1 scans to narrow down candidate peptides, finding up to 89% of the peptides identified by analogous DDA experiments. DIA-Umpire also includes additional methods that generate new reference/library spectra, incorporates prior library spectra, and uses them for further steps in protein identification and quantification, achieving on average a 0.931 R ^2^ correlation in the quantifications of peptides between replicates. This strategy works in a fashion analogous to DDA experiments in that it identifies many peptides, but also the strategy provides precise DIA quantification of the identified peptides ^35^.

### Dot-product scoring with additional heuristics

These strategies are inspired by traditional database searching and do not focus on using dynamic patterns to identify peptides. Instead, they include other heuristics to adapt searching to a DIA context. They score each peptide against each observed spectrum by computing the dot-product of the peptide’s theoretical spectrum against each observed spectrum, much like traditional DDA search algorithms. However, these methods introduce additional heuristic filtering steps, such as considering only observed spectra that match a threshold number of peaks in a theoretical spectrum. The first example of this strategy was FT-ARM ^37^. However, a more recent method, Pecan ^38^, contains additional heuristics as well as a discriminative model to combine them, so it performs better than FT-ARM. Pecan is available through the Skyline ^34– 37, 39^ graphical user interface, which provides convenient visualization, annotation, and analysis of mass spectra acquired by DDA, DIA, and MRM/PRM.

### Chromatogram scoring with spectral libraries

Strategies in this category are adapted from methods used to analyze PRM/MRM spectra, because both DIA and MRM/PRM repeatedly sample the same peptides to obtain sequences of fragment ion intensities over elution time (fragment ion chromatograms; Figure 2D). These methods take library spectra as input (compiled from prior DDA peptide identifications or prior DIA-Umpire identifications) and extract fragment ion chromatograms at the peaks in the library spectra. Potential elution peaks of each peptide from these fragment ion chromatograms are evaluated on the basis of many criteria, including how well the fragment ions correlate over elution time and how well their relative intensities match their corresponding library spectrum. Elution peaks are evaluated by using a discriminative model that combines these criteria to distinguish real peptide signals from decoy peptide signals. These methods also quantify proteins by quantifying the fragment ion chromatograms of their peptides.

OpenSWATH ^31^, SWATHProphet ^32^, Spectronaut ^40^, and a module in DIA-Umpire ^35^ all implement this strategy. Skyline ^39^ provides elution peak quantification but not statistical validation. OpenSWATH ^31^ has been shown to achieve coefficients of variation of between 0% and 20% on 345 spike-in peptides across 256-fold concentration differences in a yeast lysate. In a series of human lysate runs, Spectronaut ^40^ achieved a 98% peptide identification reproducibility rate from run to run on DIA data compared with 49% on DDA data on 26,738 peptides covering 3,690 proteins.

The class of search methods to be used depends on the specific context of the experiment. If no library spectra are available, then Pecan or DIA-Umpire must be used. No published direct comparison yet exists between the two, so the choice depends on how they fit into your bioinformatics pipeline. DIA-Umpire includes its own pipeline for library spectrum generation and automated quantification. Pecan is incorporated into Skyline ^39^ such that visualization, annotation, and semi-automated quantification are convenient. If library spectra are available, then a method with chromatogram scoring should be used. Again, no direct comparison is available, but the different chromatogram scoring algorithms are similar enough such that one can use the tool that best fits into one’s pipeline.

## Examples of studies facilitated by the increased breadth and precision of DIA

Here, we describe the use of algorithm 3, chromatogram scoring with spectral libraries, on two large quantitative studies whose insights critically depend on the breadth, reproducibility, and quantitative precision of DIA. The first study characterizes plasma proteome variation between monozygotic and dizygotic twins ^41^ and elucidates biomarker variation over time. Its inclusion of both types of twins allows the quantification of variation caused by genetics, the environment, and time. The study sampled, at two time points, blood from each person in 22 pairs of adult fraternal twins and 36 pairs of adult identical twins, resulting in 232 sets of DIA experiments. The spectral library used to identify and quantify peptides consists of 43,000 peptides that represent 1,667 unique proteins, compiled from a pre-existing library ^42^ and supplementary DDA data. OpenSWATH analysis was able to identify 1,904 of these peptides (342 proteins) in all 232 samples and quantify 76% of these with coefficients of variation of less than 25%. Notably, 42 of the identified proteins are approved by the US Food and Drug Administration for clinical assays. The breadth and precision of quantification vastly surpassed prior attempts using antibody arrays ^43^ and could be achieved only by using DIA rather than PRM or DDA.

A thorough survey of the successfully identified proteins characterized the main causes of their variability: proteins involved in blood coagulation, inflammation, and high-density lipoproteins that regulate cholesterol levels are controlled more by genetics than environmental influences. These results corroborate findings from several prior studies. Moreover, the survey discovered previously uncharacterized dependencies of eight of the clinically relevant proteins on age, which could confound their clinical interpretation. For example, plasma level of soluble CD14 is used as an independent predictor for HIV infection, and 14-3-3 protein zeta/delta is used as a prognostic for lung and breast cancers; both of these proteins naturally vary over time ^41^.

In a second study, DIA and OpenSWATH successfully mapped the interactomes of four well-characterized human proteins via affinity purification of Flag-tagged proteins and discovered how the interactions of two of them with the chaperone protein HSP90 differ in response to melanoma-associated mutations ^44^. In particular, DIA analysis of CDK4 affinity-purified samples out-identified and out-quantified analogous DDA experiments: 5,089 peptides were identified in all three DIA replicates, whereas 2,741 were identified in all three DDA replicates. Of peptides identified by both DIA and DDA, DIA quantified 82.1% with a coefficient of variation of less than 20% compared with the 74.5% achieved by DDA. Samples affinity-purified for melanoma-associated mutant versions of CDK4 showed increases in HSP90 abundance, suggesting that these mutants associate more or form a stronger association than wild-type CDK4 with HSP90.

## Future directions

Although DIA has been gradually accepted by the proteomics community, improvements in hardware and software tools are still required to facilitate its use by the larger scientific community. From a hardware standpoint, parallel improvements in duty-cycle speed, sensitivity, and peak resolution, especially at the MS2 level, will be critical for the improvement of DIA. Faster duty-cycle speed could allow increases in the mass regions analyzed by a DIA experiment in a single run. Currently, DIA experiments typically range from 100 to 400 m/z units (500 to 900 m/z region), whereas DDA usually covers 1,600 m/z units (400 to 2000 m/z region). Moreover, increases in duty cycle would allow a reduction of the size of the fragmentation windows on DIA methods from 15 to 25 m/z units on average to 2 to 5 m/z units without needing to reduce the mass range to be analyzed in a single run. Much like MRM methods, isolating small regions for fragmentation would result in increased signal-to-noise ratios at the MS2 level, which combined with increased sensitivity would allow the detection of fragment ions formerly lost in the noise. Moreover, the use of smaller fragmentation windows combined with increases in instrument resolution, currently provided by high-end instruments, would result in improved deconvolution of mixed spectra because the mixtures are simpler (fewer peptides fragmented in each MS2 event) and fragments that are close in mass are easier to differentiate (higher resolution). No mass spectrometers yet combine the resolving power and speed needed to cover the same mass range that DDA does using small windows for DIA analysis; however, mass spectrometry speed, sensitivity, and resolution have greatly improved over the last 5 years, and we are yet to reach the physical limits of hardware improvements ^45^. If the rate of instrumentation advances continues, then within the next 5 years we should be able to cover the same m/z range that DDA covers using small, overlapping DIA windows. Thus, the main challenge is how these large DIA datasets will be deconvoluted and analyzed.

To further improve the deconvolution of complex DIA spectra and increase their identification and quantification efficiency, researchers are developing more sophisticated sampling and computational algorithms to analyze biological samples using DIA. Though yet unpublished, several such methods were presented at the American Society of Mass Spectrometry conference. Three of the most promising analysis methods are outlined below.

The first method draws a parallel between the analysis of DIA spectra and the field of compressed sensing to precisely infer the precursor masses of the detected fragment ions ^46^. It uses the random, multiplexed sampling of MSX ^34^ but improves the deconvolution of multiplexed spectra by adding further constraints to the system of linear equations posed by deconvolution, inspired by the field of compressed sensing. Compressed sensing takes advantage of the fact that if a signal is sparse, then one need not measure all of the signal to accurately reconstruct it ^47^. DIA data are sparse; at any time during the chromatography, only a small fraction of possible precursor peaks is observed. To precisely match precursor ions to their fragments, it is unnecessary to dedicate fragment scans to all small individual precursor windows if most windows are devoid of peptides. Indeed, DIA methods use wide, contiguous precursor isolation windows of width up to 25 m/z. However, compressed sensing states that repeated sampling from these predetermined windows are suboptimal to match fragment ions to their precursors. Instead, if one randomly combines smaller, not necessarily adjacent windows into large composite windows that overall span 25 m/z, then one can match fragments to precursors within these smaller, more precise windows. The published MSX ^34^ method already leverages this fact but does not use the theoretical techniques developed in compressed sensing to get the most accurate deconvolution.

The second method attempts to improve identifications by using linear regression to jointly identify the whole set of present peptides simultaneously rather than one at a time ^48^. Unlike current methods, the regression can deconvolve fragment ion interference in a principled way, which occurs when multiple precursor ions contribute to intensity at the same fragment ion peak, such that its chromatographic profile no longer matches the chromatographic profiles of the other fragment ions. Estimates have shown that approximately 24% of peptides may exhibit interference in a human urine sample ^32^. This phenomenon prevents current methods from attributing the fragment ion peak to any single precursor ion, but the regression approach can in principle properly attribute the peak to all appropriate precursor ions simultaneously.

The third method extends traditional DDA search methods with peak filtering to take into account the correlation of fragment ion peaks from the same peptide ^49^. When scoring matches between candidate peptides and observed MS2 scans, some shared peaks can be explained by multiple candidate peptides and some unique peaks can be explained by just one. The method restricts each shared peak to contribute to the score of only the single peptide whose unique peaks correlate best over time with the shared peak. This restriction prevents the spurious high scores of falsely discovered peptides that depend entirely on peaks originating from other peptides. These techniques to better associate fragment ions to their precursor ions are in developmental stages and, if successful, will broaden the usefulness of any DIA dataset.

## Conclusions

Because DIA combines the breadth of protein identification provided by DDA and approaches the sensitivity and precision of MRM/PRM, it will be the best choice for discovery bottom-up proteomics and large-scale quantification in the near future. Sampling schemes and analysis methods already allow flexibility to adapt DIA to most biological problems and researcher needs. However, because DIA is still a work in progress, DDA data are still easier to acquire and analyze for most researchers and DDA is the method of choice for most biology and proteomics laboratories. Finally, using DIA implies trading off some precision and sensitivity for breadth when compared with targeted methods. Thus, if quantification of specific lowly abundant peptides is required, then MRM and PRM targeting the ions of interest are still the better option.

## Abbreviations

DDA, data-dependent acquisition; DIA, data-independent acquisition; HPLC, high performance liquid chromatography; LC-MS/MS, liquid chromatography-tandem mass spectrometry; MRM, multiple reaction monitoring; MS1, scan in which the peptide ions entering the mass spectrometer at a given time are identified; MS2, scan in which the fragments of all (or some) of the peptides that are in the mass spectrometer at a given time are identified; m/z, mass-to-charge ratio; PRM, parallel reaction monitoring.

## Supplementary material

Dataset 1. Zip file contains 4 files: 3 files that show false discovery rates and metadata for the 345 Synthetic Gold Standard peptides spiked into the yeast, human, and water backgrounds from the experiments shown in Figure 3. Each file is associated with one background and contains identifications from 30 experiments spanning 10 spike-in concentrations with 3 replicates each. The "m_score" column denotes the false discovery rates reported by OpenSWATH. The 4th file "mf_files.txt" further annotates the 90 experiments.

## References

[1] Mann M Hendrickson RC Pandey A: Analysis of proteins and proteomes by mass spectrometry. *Annu Rev Biochem.* 2001;70:437–73. 10.1146/annurev.biochem.70.1.437 11395414

[2] Ong SE Blagoev B Kratchmarova I: Stable isotope labeling by amino acids in cell culture, SILAC, as a simple and accurate approach to expression proteomics. *Mol Cell Proteomics.* 2002;1(5):376–86. 10.1074/mcp.M200025-MCP200 12118079

[3] Ross PL Huang YN Marchese JN: Multiplexed protein quantitation in *Saccharomyces cerevisiae* using amine-reactive isobaric tagging reagents. *Mol Cell Proteomics.* 2004;3(12):1154–69. 10.1074/mcp.M400129-MCP200 15385600

[4] Bateman NW Goulding SP Shulman NJ: Maximizing peptide identification events in proteomic workflows using data-dependent acquisition (DDA). *Mol Cell Proteomics.* 2014;13(1):329–38. 10.1074/mcp.M112.026500 23820513 PMC3879624

[5] Wolf-Yadlin A Hautaniemi S Lauffenburger DA: Multiple reaction monitoring for robust quantitative proteomic analysis of cellular signaling networks. *Proc Natl Acad Sci U S A.* 2007;104(14):5860–5. 10.1073/pnas.0608638104 17389395 PMC1851582

[6] Addona TA Abbatiello SE Schilling B: Multi-site assessment of the precision and reproducibility of multiple reaction monitoring-based measurements of proteins in plasma. *Nat Biotechnol.* 2009;27(7):633–41. 10.1038/nbt.1546 19561596 PMC2855883

[7] Stergachis AB MacLean B Lee K: Rapid empirical discovery of optimal peptides for targeted proteomics. *Nat Methods.* 2011;8(12):1041–3. 10.1038/nmeth.1770 22056677 PMC3227787

[8] Peterson AC Russell JD Bailey DJ: Parallel reaction monitoring for high resolution and high mass accuracy quantitative, targeted proteomics. *Mol Cell Proteomics.* 2012;11(11):1475–88. 10.1074/mcp.O112.020131 22865924 PMC3494192

[9] Venable JD Dong MQ Wohlschlegel J: Automated approach for quantitative analysis of complex peptide mixtures from tandem mass spectra. *Nat Methods.* 2004;1(1):39–45. 10.1038/nmeth705 15782151

[10] Bilbao A Varesio E Luban J: Processing strategies and software solutions for data-independent acquisition in mass spectrometry. *Proteomics.* 2015;15(5–6):964–80. 10.1002/pmic.201400323 25430050

[11] Aebersold R Mann M: Mass spectrometry-based proteomics. *Nature.* 2003;422(6928):198–207. 10.1038/nature01511 12634793

[12] Wolf-Yadlin A Kumar N Zhang Y: Effects of HER2 overexpression on cell signaling networks governing proliferation and migration. *Mol Syst Biol.* 2006;2:54. 10.1038/msb4100094 17016520 PMC1682017

[13] Argenzio E Bange T Oldrini B: Proteomic snapshot of the EGF-induced ubiquitin network. *Mol Syst Biol.* 2011;7:462. 10.1038/msb.2010.118 21245847 PMC3049407

[14] Villén J Beausoleil SA Gerber SA: Large-scale phosphorylation analysis of mouse liver. *Proc Natl Acad Sci U S A.* 2007;104(5):1488–93. 10.1073/pnas.0609836104 17242355 PMC1785252

[15] Ballif BA Villén J Beausoleil SA: Phosphoproteomic analysis of the developing mouse brain. *Mol Cell Proteomics.* 2004;3(11):1093–101. 10.1074/mcp.M400085-MCP200 15345747

[16] Blagoev B Kratchmarova I Ong SE: A proteomics strategy to elucidate functional protein-protein interactions applied to EGF signaling. *Nat Biotechnol.* 2003;21(3):315–8. 10.1038/nbt790 12577067

[17] Gavin AC Maeda K Kühner S: Recent advances in charting protein-protein interaction: mass spectrometry-based approaches. *Curr Opin Biotechnol.* 2011;22(1):42–9. 10.1016/j.copbio.2010.09.007 20934865

[18] Davis ZH Verschueren E Jang GM: Global mapping of herpesvirus-host protein complexes reveals a transcription strategy for late genes. *Mol Cell.* 2015;57(2):349–60. 10.1016/j.molcel.2014.11.026 25544563 PMC4305015

[19] Meyniel-Schicklin L de Chassey B André P: Viruses and interactomes in translation. *Mol Cell Proteomics.* 2012;11(7):M111.014738. 10.1074/mcp.M111.014738 22371486 PMC3394946

[20] Navare AT Sova P Purdy DE: Quantitative proteomic analysis of HIV-1 infected CD4+ T cells reveals an early host response in important biological pathways: protein synthesis, cell proliferation, and T-cell activation. *Virology.* 2012;429(1):37–46. 10.1016/j.virol.2012.03.026 22542004 PMC3358407

[21] Diner BA Lum KK Javitt A: Interactions of the Antiviral Factor Interferon Gamma-Inducible Protein 16 (IFI16) Mediate Immune Signaling and Herpes Simplex Virus-1 Immunosuppression. *Mol Cell Proteomics.* 2015;14(9):2341–56. 10.1074/mcp.M114.047068 25693804 PMC4563720

[22] Cristea IM Graham D: Virology meets Proteomics. *Proteomics.* 2015;15(12):1941–2. 10.1002/pmic.201570103 26082410 PMC4677572

[23] Ong SE Mann M: A practical recipe for stable isotope labeling by amino acids in cell culture (SILAC). *Nat Protoc.* 2006;1(6):2650–60. 10.1038/nprot.2006.427 17406521

[24] Wiese S Reidegeld KA Meyer HE: Protein labeling by iTRAQ: a new tool for quantitative mass spectrometry in proteome research. *Proteomics.* 2007;7(3):340–50. 10.1002/pmic.200600422 17177251

[25] Lange V Picotti P Domon B: Selected reaction monitoring for quantitative proteomics: a tutorial. *Mol Syst Biol.* 2008;4(1):222. 10.1038/msb.2008.61 18854821 PMC2583086

[26] Whiteaker JR Halusa GN Hoofnagle AN: CPTAC Assay Portal: a repository of targeted proteomic assays. *Nat Methods.* 2014;11(7):703–4. 10.1038/nmeth.3002 24972168 PMC4113142

[27] Kim YJ Gallien S van Oostrum J: Targeted proteomics strategy applied to biomarker evaluation. *Proteomics Clin Appl.* 2013;7(11–12):739–47. 10.1002/prca.201300070 24123942

[28] Tsuchiya H Tanaka K Saeki Y: The parallel reaction monitoring method contributes to a highly sensitive polyubiquitin chain quantification. *Biochem Biophys Res Commun.* 2013;436(2):223–9. 10.1016/j.bbrc.2013.05.080 23726910

[29] Koyano F Okatsu K Kosako H: Ubiquitin is phosphorylated by PINK1 to activate parkin. *Nature.* 2014;510(7503):162–6. 10.1038/nature13392 24784582

[30] Majovsky P Naumann C Lee CW: Targeted proteomics analysis of protein degradation in plant signaling on an LTQ-Orbitrap mass spectrometer. *J Proteome Res.* 2014;13(10):4246–58. 10.1021/pr500164j 25130057

[31] Röst HL Rosenberger G Navarro P: OpenSWATH enables automated, targeted analysis of data-independent acquisition MS data. *Nat Biotechnol.* 2014;32(3):219–23. 10.1038/nbt.2841 24727770

[32] Keller A Bader SL Shteynberg D: Automated Validation of Results and Removal of Fragment Ion Interferences in Targeted Analysis of Data-independent Acquisition Mass Spectrometry (MS) using SWATHProphet. *Mol Cell Proteomics.* 2015;14(5):1411–8. 10.1074/mcp.O114.044917 25713123 PMC4424409

[33] Gillet LC Navarro P Tate S: Targeted data extraction of the MS/MS spectra generated by data-independent acquisition: a new concept for consistent and accurate proteome analysis. *Mol Cell Proteomics.* 2012;11(6):O111.016717. 10.1074/mcp.O111.016717 22261725 PMC3433915

[34] Egertson JD Kuehn A Merrihew GE: Multiplexed MS/MS for improved data-independent acquisition. *Nat Methods.* 2013;10(8):744–6. 10.1038/nmeth.2528 23793237 PMC3881977

[35] Tsou CC Avtonomov D Larsen B: DIA-Umpire: comprehensive computational framework for data-independent acquisition proteomics. *Nat Methods.* 2015;12(3):258–64, 7 p following 264. 10.1038/nmeth.3255 25599550 PMC4399776

[36] Bern M Finney G Hoopmann MR: Deconvolution of mixture spectra from ion-trap data-independent-acquisition tandem mass spectrometry. *Anal Chem.* 2010;82(3):833–41. 10.1021/ac901801b 20039681 PMC2813958

[37] Weisbrod CR Eng JK Hoopmann MR: Accurate peptide fragment mass analysis: multiplexed peptide identification and quantification. *J Proteome Res.* 2012;11(3):1621–32. 10.1021/pr2008175 22288382 PMC3319072

[38] Ting YS Egertson J Maclean B: Pecan: Peptide identification directly from data-independent acquisition (DIA) MS/MS data. Poster at *ASMS*,2014.

[39] MacLean B Tomazela DM Shulman N: Skyline: an open source document editor for creating and analyzing targeted proteomics experiments. *Bioinformatics.* 2010;26(7):966–8. 10.1093/bioinformatics/btq054 20147306 PMC2844992

[40] Bruderer R Bernhardt OM Gandhi T: Extending the limits of quantitative proteome profiling with data-independent acquisition and application to acetaminophen-treated three-dimensional liver microtissues. *Mol Cell Proteomics.* 2015;14(5):1400–10. 10.1074/mcp.M114.044305 25724911 PMC4424408

[41] Liu Y Buil A Collins BC: Quantitative variability of 342 plasma proteins in a human twin population. *Mol Syst Biol.* 2015;11(1):786. 10.15252/msb.20145728 25652787 PMC4358658

[42] Farrah T Deutsch EW Omenn GS: A high-confidence human plasma proteome reference set with estimated concentrations in PeptideAtlas. *Mol Cell Proteomics.* 2011;10(9):M110.006353. 10.1074/mcp.M110.006353 21632744 PMC3186192

[43] Kato BS Nicholson G Neiman M: Variance decomposition of protein profiles from antibody arrays using a longitudinal twin model. *Proteome Sci.* 2011;9:73. 10.1186/1477-5956-9-73 22093360 PMC3247853

[44] Lambert JP Ivosev G Couzens AL: Mapping differential interactomes by affinity purification coupled with data-independent mass spectrometry acquisition. *Nat Methods.* 2013;10(12):1239–45. 10.1038/nmeth.2702 24162924 PMC3882083

[45] Richards AL Merrill AE Coon JJ: Proteome sequencing goes deep. *Curr Opin Chem Biol.* 2015;24:11–7. 10.1016/j.cbpa.2014.10.017 25461719 PMC4308434

[46] Egertson J Johnson RS Xuan Y: Improved Computational Demultiplexing for Data Independent Acquisition Data Acquired by MSX or with Overlapping Windows. Presentation at *ASMS*,2015.

[47] Mackenzie D: Compressed Sensing Makes Every Pixel Count. *What's Happening in the Mathematical Sciences* Providence, RI: American Mathematical Society;2009;115–27. Reference Source

[48] Hu A Howbert JJ Bilmes J: A regularized linear regression model for the identification of peptides from data-independent acquisition mass spectra.Poster at *ASMS*,2015.

[49] Afkham HM Kim S Käll L: Improving DIA peptide identification via local time profile similarity. Poster at *ASMS*,2015.

[50] Egertson JD MacLean B Johnson R: Multiplexed peptide analysis using data-independent acquisition and Skyline. *Nat Protoc.* 2015;10(6):887–903. 10.1038/nprot.2015.055 25996789 PMC5127711

[51] Deutsch EW Mendoza L Shteynberg D: A guided tour of the Trans-Proteomic Pipeline. *Proteomics.* 2010;10(6):1150–9. 10.1002/pmic.200900375 20101611 PMC3017125

[52] McIlwain S Tamura K Kertesz-Farkas A: Crux: rapid open source protein tandem mass spectrometry analysis. *J Proteome Res.* 2014;13(10):4488–91. 10.1021/pr500741y 25182276 PMC4184452

[53] Tabb DL Vega-Montoto L Rudnick PA: Repeatability and reproducibility in proteomic identifications by liquid chromatography-tandem mass spectrometry. *J Proteome Res.* 2010;9(2):761–76. 10.1021/pr9006365 19921851 PMC2818771

[54] Liebler DC Zimmerman LJ: Targeted quantitation of proteins by mass spectrometry. *Biochemistry.* 2013;52(22):3797–806. 10.1021/bi400110b 23517332 PMC3674507

